# AMSTAR 2 appraisal of systematic reviews and meta-analyses in the field of heart failure from high-impact journals

**DOI:** 10.1186/s13643-022-02029-9

**Published:** 2022-07-23

**Authors:** Lin Li, Iriagbonse Asemota, Bolun Liu, Javier Gomez-Valencia, Lifeng Lin, Abdul Wahab Arif, Tariq Jamal Siddiqi, Muhammad Shariq Usman

**Affiliations:** 1 Department of Medicine, Cook County Health, Chicago, IL USA; 2 Division of Cardiology, Cook County Health, Rush University Medical Center, Chicago, IL USA; 3 Department of Statistics, Florida State University, Tallahassee, Florida USA; 4 Department of Medicine, Dow University of Health Sciences, Karachi, Pakistan

**Keywords:** AMSTAR 2, Heart failure, Meta-analysis, Systematic review

## Abstract

**Background:**

The Measurement Tool to Assess systematic Reviews (AMSTAR) 2 is a critical appraisal tool for systematic reviews (SRs) and meta-analyses (MAs) of interventions. We aimed to perform the first AMSTAR 2-based quality assessment of heart failure-related studies.

**Methods:**

Eleven high-impact journals were searched from 2009 to 2019. The included studies were assessed on the basis of 16 domains. Seven domains were deemed critical for high-quality studies. On the basis of the performance in these 16 domains with different weights, overall ratings were generated, and the quality was determined to be “high,” “moderate,” “low,” or “critically low.”

**Results:**

Eighty-one heart failure-related SRs with MAs were included. Overall, 79 studies were of “critically low quality” and two were of “low quality.” These findings were attributed to insufficiency in the following critical domains: a priori protocols (compliance rate, 5%), complete list of exclusions with justification (5%), risk of bias assessment (69%), meta-analysis methodology (78%), and investigation of publication bias (60%).

**Conclusions:**

The low ratings for these potential high-quality heart failure-related SRs and MAs challenge the discrimination capacity of AMSTAR 2. In addition to identifying certain areas of insufficiency, these findings indicate the need to justify or modify AMSTAR 2’s rating rules.

**Supplementary Information:**

The online version contains supplementary material available at 10.1186/s13643-022-02029-9.

## Introduction

Since the 1970s, systematic reviews (SRs) and meta-analyses (MAs) began to gain prominence as the science of research synthesis began and play a role in providing evidence-based information to drive decision making. These publications are now widely used in clinical and policy decisions, making it imperative to verify their quality. A high-quality study follows standard protocols and reports relevant details at every step to facilitate readers’ understanding of its results. Various guidelines have been proposed for SRs and MAs, including the Cochrane Handbook, Preferred Reporting Items for Systematic Reviews and Meta-Analyses (PRISMA), Quality of Reporting of Meta-analyses (QUORUM), and Meta-analyses Of Observational Studies in Epidemiology (MOOSE) [1–4]. Appraisal tools such as the Measurement Tool to Assess systematic Reviews (AMSTAR); AMSTAR 2; revised AMSTAR (R-AMSTAR); Scottish Intercollegiate Guidelines Network (SIGN) checklist; Grading of Recommendations, Assessment, Development and Evaluations (GRADE) framework; and Risk of Bias Assessment Tool for Systematic Reviews (ROBIS) have also been developed to evaluate these studies [5–10].

AMSTAR was developed in 2007 on the basis of the Cochrane Handbook for Systematic Reviews of Interventions [1, 5]. It primarily focuses on the correct methodology to ensure reliable results and serves as a brief checklist of items needed for high-quality reviews. After a decade of extensive use, an updated version, AMSTAR 2, based on users’ experience and feedback, was published in 2017 [6]. AMSTAR 2 is a domain-based rating system with seven critical domains and nine non-critical domains. Instead of generating a total score, AMSTAR 2 evaluates the overall quality based on performance in critical and non-critical domains, which are assigned different weights in the rating rules. As a part of the advancements from AMSTAR, which only appraises SRs based on randomized controlled trials (RCTs), AMSTAR 2 also appraises SRs of non-randomized studies of interventions (NRSIs) using specific contents covering a different risk of bias (RoB) assessment and meta-analysis methodology. AMSTAR 2 also tightens the rules for appraisal of each domain by retaining only “yes” or “no” responses and removing “not applicable” and “cannot answer” responses. Since 2017, approximately 100 publications have used AMSTAR 2 to evaluate SRs in different areas [11–17]. However, reports using this tool in the field of heart failure (HF) have been limited. Therefore, we identified HF-related SRs and MAs from high-impact journals over the last 10 years and assessed their quality using AMSTAR 2.

## Methods

### Data source and study selection

A systematic search was conducted in August 2019 by two independent reviewers (Lin Li and Abdul Wahab Arif to identify all HF-related SRs and MAs published between January 2009 and July 2019 in 11 major medical and cardiovascular journals: *Annals of Internal Medicine* (Annals IM), *Circulation*, *Circulation: Heart Failure*, *European Heart Journal* (EHJ), *European Journal of Heart Failure* (EJHF), *Journal of American College of Cardiology* (JACC), *Journal of American College of Cardiology: Heart Failure* (JACC HF), *Journal of American Medical Association* (JAMA), *Journal of American Medical Association: Internal Medicine* (JAMA-IM), *Lancet*, and *the New England Journal of Medicine* (NEJM). These journals were selected based on their high impact factors and their focus on publishing high-quality SRs and MAs related to HF.

We performed manual searches for the terms “meta” and “review” in the titles and abstracts on the journals’ websites and excluded papers that did not include MAs and those unrelated to HF. Because AMSTAR 2 was designed for the SRs and MAs of healthcare interventions, we further excluded papers focused on epidemiology, diagnosis, prognosis, and etiology. Moreover, AMSTAR 2 was not intended to deal with MAs of individual patient data or network MAs, so such papers were also excluded.

### Data extraction and quality assessment by AMSTAR 2

Two authors (Lin Li and Abdul Wahab Arif) independently reviewed the full texts and Online supplemental material of each included study. We extracted information, including PubMed ID (PMID), publication year, journal, authors, type of study (RCT- or NRSI-based), Cochrane or non-Cochrane SR, and number of citations.

Two authors (Lin Li and Iriagbonse Asemota) independently used the AMSTAR 2 online checklist to evaluate each included study [18]. Disagreements were resolved by a third reviewer, Lifeng Lin, while referring to AMSTAR 2’s original paper, supplements, and the relevant chapters in the Cochrane Handbook [1, 6]. The seven critical domains and nine non-critical domains are listed in Table 1. We recorded the answers to all 16 domains. All answers were categorized as “yes” or “no” in AMSTAR 2. “Partial yes” was allowed in some domains to identify partial adherence to standard protocols. If the answer was “no” to a specific domain, this domain was labeled as “weak.”

**Table 1:** Sixteen domains in AMSTAR 2

Domain number	Critical or non-critical	Content of the domain	Yes or partial yes (%)	No (%)
1	Non-critical domain	Did the research questions and inclusion criteria for the review include the components of PICO^a^?	65	35
2	Critical domain	Did the report of the review contain an explicit statement that the review methods were established prior to the conduct of the review and did the report justify any significant deviations from the protocol?	5	95
3	Non-critical domain	Did the review authors explain their selection of the study designs for inclusion in the review?	15	85
4	Critical domain	Did the review authors use a comprehensive literature search strategy?	86	14
5	Non-critical domain	Did the review authors perform study selection in duplicate?	68	32
6	Non-critical domain	Did the review authors perform data extraction in duplicate?	73	27
7	Critical domain	Did the review authors provide a list of excluded studies and justify the exclusions?	5	95
8	Non-critical domain	Did the review authors describe the included studies in adequate detail?	83	17
9	Critical domain	Did the review authors use a satisfactory technique for assessing the risk of bias in individual studies that were included in the review?	69	31
10	Non-critical domain	Did the review authors report on the sources of funding for the studies included in the review?	15	85
11	Critical domain	If meta-analysis was performed did the review authors use appropriate methods for statistical combination of results?	78	22
12	Non-critical domain	If meta-analysis was performed, did the review authors assess the potential impact of risk of bias in individual studies on the results of the meta-analysis or other evidence synthesis?	27	73
13	Critical domain	Did the review authors account for risk of bias in individual studies when interpreting/ discussing the results of the review?	88	12
14	Non-critical domain	Did the review authors provide a satisfactory explanation for, and discussion of, any heterogeneity observed in the results of the review?	85	15
15	Critical domain	If they performed quantitative synthesis did the review authors carry out an adequate investigation of publication bias (small study bias) and discuss its likely impact on the results of the review?	60	40
16	Non-critical domain	Did the review authors report any potential sources of conflict of interest, including any funding they received for conducting the review?	99	1

^a^*PICO* Population, Intervention, Control, and Outcomes

Subsequently, we rated the studies as high-, moderate-, low-, or critically low-quality. The rules of the overall rating based on these 16 domains are listed below [6].

### High

No or one non-critical weakness: The systematic review provides an accurate and comprehensive summary of the results of the available studies that address the question of interest.

### Moderate

More than one non-critical weakness*: The systematic review has more than one weakness but no critical flaws. This may provide an accurate summary of the results of the available studies included in the review.

### Low

One critical flaw with or without non-critical weaknesses: The review has a critical flaw and may not provide an accurate and comprehensive summary of the available studies that address the question of interest.

### Critically low

More than one critical flaw with or without non-critical weaknesses: The review has more than one critical flaw and should not be relied on to provide an accurate and comprehensive summary of the available studies.

*Multiple non-critical weaknesses may diminish confidence in the review, and it may be appropriate to move the overall appraisal down from moderate to low confidence.

We explored the inter-rater reliability (IRR) by using Cohen’s kappa coefficient for each domain. A value < 0 indicates no agreement; 0–0.20, slight agreement; 0.21–0.40, fair agreement; 0.41–0.60, moderate agreement; 0.61–0.80, substantial agreement; and 0.81–1, almost perfect agreement [19].

## Results

### Literature search and study characteristics

**Fig. 1:**
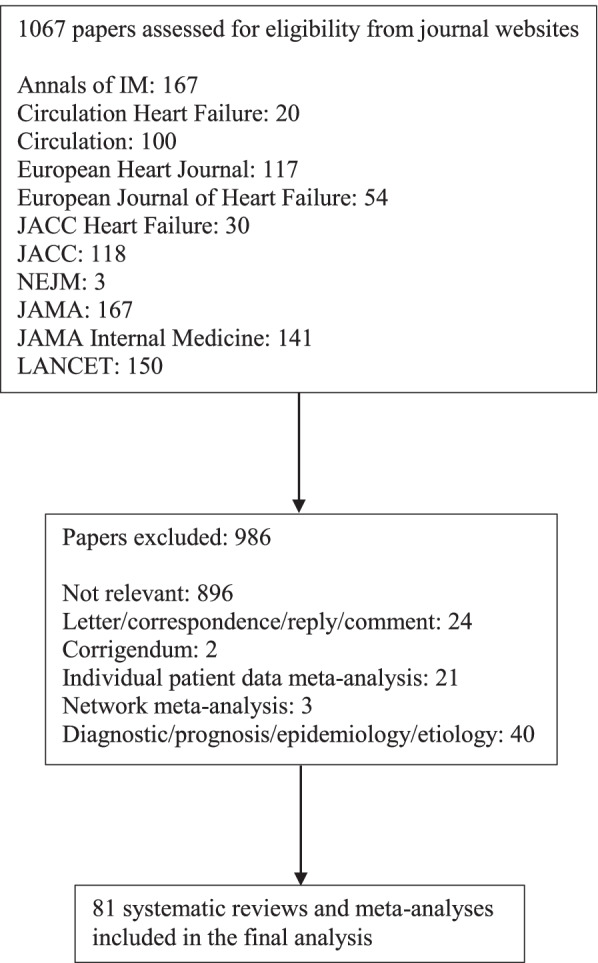
Flow chart

We included 81 SRs and MAs on HF-related interventions from eight journals (Fig. 1). None of the articles from NEJM, Lancet, and JAMA were included due to the aforementioned inclusion and exclusion criteria. Of the 81 articles, 56 studies were based on RCTs, 13 were based on NRSIs, and 12 included both RCTs and NRSIs. Nine studies were Cochrane systematic reviews, whereas 72 studies were non-Cochrane reviews. As of July 2019, the average number of citations was 104 (Online Supplement 1).

### Results for the 16 domains

The results for each domain are presented as percentages of “yes or partial yes” and “no” answers, as detailed in Table 1.

For Q2, only 4 (5%) studies answered “yes,” and they were either registered in PROSPERO or published pre-established protocols. Among the other 77 studies, 43 did not report any guidelines or protocols. The remaining 34 studies followed guidelines such as the Center for Reviews and Dissemination (CRD), PRISMA, Cochrane Handbook, QUOROM, and MOOSE [1–4, 20].

The answer for Q9 was “no” in 25 (31%) studies because the RoB assessment was not satisfactory. For RCT-based studies, most of the “no” answers were attributed to the lack of RoB assessment tools, for which the most commonly used appropriate tools were the Cochrane instrument and JADAD [21, 22]. Similarly, for the NRSI-based studies, most of the “no” answers were attributed to the lack of reporting tools. The most commonly used tools were the Risk Of Bias In Non-randomized Studies-of Interventions (ROBINS), Strengthening the Reporting of Observational Studies in Epidemiology (STROBE), Newcastle-Ottawa Scale (NOS), and United States Preventive Services Task Force (USPSTF) checklists [23–26]. For studies based on both RCTs and NRSIs, the insufficiency was caused by the fact that one tool covered RCTs but failed to cover NRSIs, or vice versa. The appropriate tools reported in these studies that can cover both RCTs and NRSIs were the Cochrane instrument and Downs and Black checklist [1, 27]. The answer for Q12 was “no” in 59 (73%) studies. This is another RoB-related domain that examines how results vary with inclusion or exclusion of studies with a high RoB [6]. Among the 22 studies with “yes” answers to Q12, 13 had low RoB, six used sensitivity analyses, two used subgroup analyses, and one used meta-regression analyses.

The answer for Q11 was “no” in 18 (22%) studies. One of the 56 RCT-based MAs failed Q11 because it included no investigation of heterogeneity. Seven of the 13 NRSI-based MAs failed Q11 because they did not report confounder-adjusted estimates and instead used raw data for pooling. Among the 12 studies based on both RCTs and NRSIs, 10 failed Q11 because of failure to clarify the NRSI data source or failure to report summaries separately for RCTs and NRSIs. The answer for Q14 was “no” in 12 (15%) studies due to a lack of a satisfactory explanation for heterogeneity. Nineteen studies reported no significant heterogeneity, and 17 used meta-regression analyses. Nine used subgroup analyses, eight used sensitivity analyses, and 16 explained the heterogeneity narratively.

Cohen’s kappa varied among the 16 domains, but most of the values were in the range of acceptable agreement. Fair, moderate, substantial, and perfect agreement were recorded for three (Q1, Q12, and Q13), three (Q8, Q9, and Q14), four (Q3, Q5, Q10, and Q11), and eight (Q2, Q4, Q6, Q7, Q15, and Q16) domains, respectively (Online Supplement 2).

### Overall ratings

Although AMSTAR 2 stresses that it is not intended to generate an overall score, we completed the assessment process and rated these 81 studies as two low-quality reviews and 79 critically low-quality reviews following the rules mentioned above.

## Discussion

Based on our literature review from 2009 to 2019, this is the first study to apply AMSTAR 2 in highly cited HF-related SRs and MAs. Sixteen domains evaluated each step of the conduct of SRs and MAs. AMSTAR 2 has been previously used in various fields, including psychiatry, surgery, pediatrics, endocrinology, rheumatology, and cardiovascular disease, and many publications have reported substantial numbers of SRs with low to critically low quality [28–33]. Shan Shan et al. in prior literature called it a “floor effect” because of the lack of discrimination capacity of the tool, raising questions about its high standard and practical value [34]. In this study, we observed similar findings for high-impact SRs and MAs related to HF. This raises the dilemma that either the AMSTAR 2 standard is unreasonable, or that these high-impact SRs and MAs are of low quality. We can further break down this question into whether AMSTAR 2’s 16 domains and its backbone Cochrane guidelines are impractical, whether AMSTAR 2’s overall rating rules are unreasonable, whether there were actual defects in these SRs and MAs, and whether there was under-reporting that should be formalized to cover the information gap and facilitate readers’ understanding of these studies.

The guidelines and evaluation systems for SRs and MAs are a rapidly evolving field, with new or updated guides released every few years [1–7, 10, 20, 35–39]. AMSTAR 2 was published in 2017, when most SRs were using the Cochrane, QUORUM, PRISMA, and MOOSE guidelines. Until 2020, less than 200 publications in PubMed used AMSTAR 2. In our review, none of the 81 studies used AMSTAR 2 as a guide. Therefore, some non-compliance can be attributed to the fact that these guidelines did not follow AMSTAR 2 from the beginning to conduct and report the study. Additionally, the strict “yes or no” rule in AMSTAR 2 precluded responses like “not applicable,” “cannot answer,” or “not reported,” forcing the evaluators to choose “no” for potentially under-reporting studies and categorize certain domains as “weak.” This probably explains why the results of quality assessment by AMSTAR 2 generally appear worse than those performed by AMSTAR in previous reviews [15].

The 95% “no” rate for the critical domains Q2 and Q7 played a major role in the overall low rating of these 81 studies, since failing one critical domain resulted in an overall low quality. These two critical domains were included since the AMSTAR in 2007. The Cochrane Handbook mentions that “All Cochrane reviews must have a written protocol, specifying in advance the scope and methods to be used by the review, to assist in planning and reduce the risk of bias in the review process.” [1] However, none of the nine Cochrane SRs included here reported pre-established protocols. PRISMA checklist also mentions, “If registered, provide the name of the registry (such as PROSPERO) and registration number,” suggesting that registration is not compulsory. Unsurprisingly, none of the 19 studies using the PRISMA checklist reported pre-established protocols. The same is true for MOOSE and QUORUM. Apparently, pre-registration or publishing a pre-established protocol is not yet a common practice for SRs, but more studies have been following this step in recent years [40–43]. Q7 is based on the same content as in Cochrane Handbook Chapter 4, with the further instruction that “the list of excluded studies should be as brief as possible. It should not list all of the reports that were identified by an extensive search.” [1] However, a complete list of exclusions is not mandated by the PROSPERO guidelines [44], nor in PRISMA or QUORUM. Although exclusion is an essential step during study selection in SRs and MAs, the various guidelines have not arrived at a consensus regarding reporting a complete list of exclusions, and there is potential difficulty for publication [45]. Thus, the designation of Q2 and Q7 as critical domains and the decision to assign an overall poor quality to studies failing them needs to be justified.

Almost half of the SRs are now based on NRSIs; an increasing number of NRSIs are based on large databases that provide a better real-world picture. Although these studies can be more precise, they are also more easily confounded [46]. Thus, one of the major advancements in AMSTAR 2 from AMSTAR is the inclusion of different RoB assessments and data-pooling methods for NRSIs. The 31% “no” answers for Q9 resulted from the lack of reporting tools or incorrectly used tools. Choosing the right RoB tool for RCTs and NRSIs remains a big challenge for authors of SRs because of the large number of tools available [47]. The lack of homogeneity among various tools also makes comparisons difficult. The RoB assessment tool for RCTs recommended by AMSTAR 2 is the Cochrane Collaboration tool, and that for NRSIs is ROBINS-I [1, 23]. In our study, different tools were eligible for the Q9 assessment. However, the same RCT or NRSI study rated as showing a low RoB by one tool may be rated as showing an elevated RoB by another more comprehensive tool. This discrepancy affected the RoB-related domains Q12 and Q13, and eventually led to different overall quality ratings, raising questions regarding AMSTAR 2’s reliability and consistency. The non-critical domain Q12 is also mentioned in the Cochrane Handbook and PROSPERO registration; however, there is no consensus regarding analysis of the influence of RoB in other checklists [1–4, 44]. Q11 further clarifies the difference between RCTs and NRSIs in terms of data-pooling methodology. The rationale for enhanced pooling of fully adjusted estimates in NRSIs is that the data adjusted by confounders may generate very different results in comparison with raw data. Deficiencies in heterogeneity investigations were another finding for Q11, which are detailed in a recent paper reviewing HF-related MAs [48]. However, the multiple domains assessing RoB and NRSI reflect their overall importance in SRs and MAs. Future researchers should better understand the differences in RoB between RCTs and NRSIs, choose RoB tools wisely, address their influence objectively, and avoid using raw data from NRSIs for meta-analysis.

The final underperforming critical domain was Q15, which covers the investigation of publication bias. Funnel plots are the best known and most commonly used method to assess publication bias or small-study effects, but at least 10 studies are needed to reliably show funnel plot asymmetry [1, 49]. Most of the SRs answered “no” for Q15 because they included less than 10 studies and did not report graphical or statistical tests of publication bias. This issue in AMSTAR 2 may need to be addressed in a future study.

In summary, AMSTAR 2 is an appraisal tool based on the Cochrane Handbook that focuses on the conduct of SRs and MAs in healthcare interventions. Our evaluation of highly cited HF-related SRs and MAs by using AMSTAR 2 helped us identify areas of insufficiency and highlighted the scope for improvements in future studies, including a priori protocol or pre-registration, the addition of a full exclusion list with justifications, appropriate RoB assessments, and caution while combining NRSI data. These findings reflect the core values of the AMSTAR 2 and Cochrane guidelines in avoiding bias. However, compared to the most commonly used guidelines mentioned above, AMSTAR 2 is relatively new and advanced. Thus, consensus among various guidance and assessment tools is essential before it can be considered as the standard. Using a “new” tool to judge older SRs and call them “low quality” is not the conclusion of this study. However, a perfect tool does not exist. AMSTAR 2 does not include justifications for designating certain domains as critical and others as non-critical and does not explain the underlying rules used to categorize studies as “high” versus “low” quality. Considering these aspects, the interaction between AMSTAR 2 and these high-impact SRs yielded some findings of interest, which can be expected to facilitate the validation of AMSTAR 2 and provide feedback for its future development.

### Limitations

We thoroughly searched all published materials related to these 81 studies, but we did not contact the review authors to clarify the “cannot answer” domains or “not reported” contents. If we had done so, some answers would have changed because of the potential underreporting in some domains, particularly Q7 and Q10. We also did not change certain non-critical domains to critical domains and vice versa for different studies, as allowed by AMSTAR 2. However, we did not include MAs that summarize the known literature base or SRs without MAs, which may question the critical nature of Q4, Q7, Q11, and Q15.

## Supplementary Information

**Additional file 1: Table 1.** Characteristics of 81 systemic reviews and meta-analyses. **Supplement Table 2.** Inter-rater reliability analysis.
