# Supplementary material for: AMSTAR 2 appraisal of systematic reviews and meta-analyses in the field of heart failure from high-impact journals
Source: Syst Rev. 2022 Jul 23;11:147. doi: 10.1186/s13643-022-02029-9 (PMC9308914; doi:10.1186/s13643-022-02029-9)
Supplement: Supplementary file 1 — Additional file 1: Table 1. Characteristics of 81 systemic reviews and meta-analyses. Supplement Table 2. Inter-rater reliability analysis. [file 13643_2022_2029_MOESM1_ESM.pdf]

SUPPLEMENTAL MATERIAL

Supplement Table I: Characteristics of 81 systemic reviews and meta-analyses

PMID	JOURNAL	YEAR	AUTHORS	INCLUDED STUDY TYPE	COCHRANE SR* OR NOT	NUMBER OF CITATION
19487713	Annals of Internal Medicine	2009	Finlay A McAlister et al.	RCT†	No	509
21041579	Annals of Internal Medicine	2010	Pasquale Santangeli et al.	RCT	No	138
21320922	Annals of Internal Medicine	2011	Nawaf S Al-Majed et al.	RCT	No	135
24592496	Annals of Internal Medicine	2014	Amy Earley et al.	RCT + NRSI‡	No	33
24862840	Annals of Internal Medicine	2014	Cynthia Feltner et al.	RCT	No	414
29404591	Annals of Internal Medicine	2017	Michalina Kolodziejczak et al.	RCT	No	26
30583296	Annals of Internal Medicine	2019	Mohit K Turagam et al.	RCT	No	13
21705485	Circulation: Heart Failure	2011	Benjamin S Wessler et al.	RCT	No	32
22821634	Circulation: Heart Failure	2012	Arend F L Schinkel et al.	NRSI	No	117
22740040	Circulation: Heart Failure	2012	Elizabeth Mostofsky et al.	NRSI	No	133
22511747	Circulation: Heart Failure	2012	Mustafa Toma et al.	RCT	No	148
23264446	Circulation: Heart Failure	2013	Meng Lee et al.	RCT	No	16
23508758	Circulation: Heart Failure	2013	Dean T Eurich et al.	NRSI	No	188
23400891	Circulation: Heart Failure	2013	Xiaobo Li et al.	RCT	No	28
23403436	Circulation: Heart Failure	2013	Srinivas R Bapoje et al.	RCT	No	36
25399909	Circulation: Heart Failure	2015	Ambarish Pandey et al.	RCT	No	215
26175539	Circulation: Heart Failure	2015	Justin B Echouffo-Tcheugui et al.	NRSI	No	34
26185169	Circulation: Heart Failure	2015	Ambarish Pandey et al.	RCT + NRSI	No	59
27729391	Circulation: Heart Failure	2016	Ajay Vallakati et al.	RCT + NRSI	No	18
28087687	Circulation: Heart Failure	2017	Georg Wolff et al.	RCT + NRSI	No	33
28790052	Circulation: Heart Failure	2017	Muhammad Shahzeb Khan et al.	RCT	No	11
28209765	Circulation: Heart Failure	2017	Iris E Beldhuis et al.	RCT	Yes	25
26438781	Circulation	2015	Ambarish Pandey et al.	RCT + NRSI	No	105
27993908	Circulation	2017	Harsh Golwala et al.	RCT	No	62
19066207	European Heart Journal	2009	Justin A Ezekowitz et al.	RCT	No	194
19168534	European Heart Journal	2009	Paolo Verdecchia et al.	RCT	No	70
19233857	European Heart Journal	2009	Mahboob Alam et al.	NRSI	No	145
19336434	European Heart Journal	2009	Jonathan P Piccini et al.	RCT	No	172
19617601	European Heart Journal	2009	Jin M Cheng et al.	RCT	Yes	457

23264584	European Heart Journal	2013	Sachin Nayyar et al.	NRSI	No	59
24026778	European Heart Journal	2014	Ronak Delewi et al.	RCT	No	122
25939649	European Heart Journal	2015	Mate Vamos et al.	RCT + NRSI	No	174
25802390	European Heart Journal	2015	David Preiss et al.	RCT	No	87
28329280	European Heart Journal	2017	Matthew J Shun-Shin et al.	RCT	No	49
19654139	European Journal of Heart Failure	2009	Susan J Brunskill et al.	RCT	Yes	73
20335354	European Journal of Heart Failure	2010	Steven A Lubitz et al.	RCT	No	57
20031949	European Journal of Heart Failure	2010	Bo Jin et al.	RCT	No	38
20525985	European Journal of Heart Failure	2010	Akshay Desai et al.	RCT	Yes	40
20494922	European Journal of Heart Failure	2010	Edward J Davies et al.	RCT	No	285
21478241	European Journal of Heart Failure	2011	Vijayalakshmi Kunadian et al.	NRSI	No	54
21193439	European Journal of Heart Failure	2011	Catherine Klersy et al.	RCT	Yes	122
21551162	European Journal of Heart Failure	2011	Ana C Alba et al.	NRSI	No	37
21733889	European Journal of Heart Failure	2011	Sally C Inglis et al.	RCT	No	336
22065869	European Journal of Heart Failure	2012	Hendrik Zimmet et al.	RCT	No	138
22510423	European Journal of Heart Failure	2012	Giuseppe Boriani et al.	RCT	No	47
22348897	European Journal of Heart Failure	2012	Tomer Avni et al.	RCT	No	114
24259043	European Journal of Heart Failure	2013	S M Afzal Sohaib et al.	RCT	No	32
23099355	European Journal of Heart Failure	2013	Lei Pan et al.	RCT	No	70
23143796	European Journal of Heart Failure	2013	Ingrid Hopper et al.	RCT	No	27
24453097	European Journal of Heart Failure	2014	Hannah Clark et al.	RCT	No	73
24797230	European Journal of Heart Failure	2014	Rong-Hui Tu et al.	RCT	No	61
24464734	European Journal of Heart Failure	2014	Xiaojing Wu et al.	RCT	No	23
25598021	European Journal of Heart Failure	2015	Susanna C Larsson et al.	NRSI	No	55
26335355	European Journal of Heart Failure	2015	Qinmei Xiong et al.	RCT	No	68
26817628	European Journal of Heart Failure	2016	Catherine Klersy et al.	RCT	No	39
27364182	European Journal of Heart Failure	2016	Scott D Solomon et al.	RCT	No	29
26821594	European Journal of Heart Failure	2016	Ewa A Jankowska et al.	RCT	No	141
27121474	European Journal of Heart Failure	2016	Ben Clevenger et al.	RCT	No	29
28597606	European Journal of Heart Failure	2017	Sameer Zaman et al.	RCT	No	7
27634736	European Journal of Heart Failure	2017	Philip B Adamson et al.	RCT + NRSI	No	22
19850208	JACC	2009	Catherine Klersy et al.	RCT + NRSI	No	347
20170823	JACC	2010	Shikhar Agarwal et al.	NRSI	No	238

20650361	JACC	2010	Daniel G Kramer et al.	RCT	Yes	236
21492765	JACC	2011	David J Holland et al.	RCT + NRSI	No	133
21851882	JACC	2011	Selcuk Adabag et al.	RCT	Yes	86
22381427	JACC	2012	Lei Zhang et al.	RCT	Yes	177
25983009	JACC	2015	Nirmalatiban Parthiban et al.	RCT	No	106
24621795	JACC: Heart Failure	2013	Michiel Rienstra et al.	RCT	No	136
24622004	JACC: Heart Failure	2013	Hashbullah Ismail et al.	RCT	No	115
26251094	JACC: Heart Failure	2015	Kurt W Prins et al.	RCT + NRSI	No	67
26454847	JACC: Heart Failure	2015	Max Liebregts et al.	NRSI	No	83
27395347	JACC: Heart Failure	2016	Kairav Vakil et al.	NRSI	No	35
27614940	JACC: Heart Failure	2016	Gianluigi Savarese et al.	RCT	No	38
30007556	JACC: Heart Failure	2018	Tiana Nizamic et al.	RCT + NRSI	No	11
30098968	JACC: Heart Failure	2018	Gabriel A Hernandez et al.	NRSI	No	2
30196071	JACC: Heart Failure	2018	Caroline K Kramer et al.	RCT	No	8
29957192	JACC: Heart Failure	2018	Oriana Ciani et al.	RCT	Yes	6
31302050	JACC: Heart Failure	2019	Rod S Taylor et al.	RCT	No	2
19752408	JAMA Internal Medicine	2009	Hamid Ghanbari et al.	RCT	No	150
20308637	JAMA Internal Medicine	2010	Pramote Porapakkham et al.	RCT	No	362
28558095	JAMA Internal Medicine	2017	Teryl K Nuckols et al.	RCT + NRSI	No	31

*SR: Systematic Review

†RCT: Randomized Controlled Trials

‡NRSI: Non-Randomized Studies of Interventions

Supplement Table II: Inter-Rater Reliability Analysis

DOMAIN	COHEN KAPPA	95% CONFIDENCE INTERVAL	OBSERVED AGREEMENT	EXPECTED AGREEMENT
1	0.38	0.19, 0.57	77%	62%
2	1.00	1.00, 1.00	100%	91%
3	0.75	0.54, 0.96	94%	76%
4	0.54	0.45, 0.61	84%	65%
5	0.70	0.54, 0.87	88%	58%
6	0.81	0.66, 0.96	93%	62%
7	1.00	1.00, 1.00	100%	91%
8	0.56	0.53, 0.69	79%	52%
9	0.45	0.39, 0.52	62%	31%
10	0.76	0.57, 0.96	94%	74%
11	0.77	0.59, 0.94	93%	68%
12	0.38	0.21, 0.55	68%	49%
13	0.39	0.12, 0.66	84%	74%
14	0.51	0.23, 0.79	89%	74%
15	0.97	0.92, 1.00	99%	52%
16	1.00	1.00, 1.00	100%	98%
